# Strategic Screening and Characterization of the Visual GPCR-mini-G Protein Signaling Complex for Successful Crystallization

**DOI:** 10.3791/60747

**Published:** 2020-03-16

**Authors:** Filip Pamula, Jonas Mühle, Alain Blanc, Rony Nehmé, Patricia C. Edwards, Christopher G. Tate, Ching-Ju Tsaic

**Affiliations:** 1 Laboratory of Biomolecular Research, Paul Scherrer Institute; 2 Department of Biology, ETH Zürich; 3 Center for Radiopharmaceutical Sciences, Paul Scherrer Institute; 4 Laboratory of Molecular Biology, Medical Research Council

**Keywords:** Biochemistry, Issue 157, detergent, purification, rhodopsin-mini-G_o_ complex, stability, glycosylation, retinal, SEC, UV-VIS spectroscopy, SDS-PAGE, LC-MS

## Abstract

The key to determining crystal structures of membrane protein complexes is the quality of the sample prior to crystallization. In particular, the choice of detergent is critical, because it affects both the stability and monodispersity of the complex. We recently determined the crystal structure of an active state of bovine rhodopsin coupled to an engineered G protein, mini-G_o_, at 3.1 Å resolution. Here, we detail the procedure for optimizing the preparation of the rhodopsin–mini-G_o_ complex. Dark-state rhodopsin was prepared in classical and neopentyl glycol (NPG) detergents, followed by complex formation with mini-G_o_ under light exposure. The stability of the rhodopsin was assessed by ultraviolet-visible (UV-VIS) spectroscopy, which monitors the reconstitution into rhodopsin of the light-sensitive ligand, 9-cis retinal. Automated size-exclusion chromatography (SEC) was used to characterize the monodispersity of rhodopsin and the rhodopsin–mini-G_o_ complex. SDS-polyacrylamide electrophoresis (SDS-PAGE) confirmed the formation of the complex by identifying a 1:1 molar ratio between rhodopsin and mini-G_o_ after staining the gel with Coomassie blue. After cross-validating all this analytical data, we eliminated unsuitable detergents and continued with the best candidate detergent for large-scale preparation and crystallization. An additional problem arose from the heterogeneity of N-glycosylation. Heterologously-expressed rhodopsin was observed on SDS-PAGE to have two different N-glycosylated populations, which would probably have hindered crystallogenesis. Therefore, different deglycosylation enzymes were tested, and endoglycosidase F1 (EndoF1) produced rhodopsin with a single species of N-glycosylation. With this strategic pipeline for characterizing protein quality, preparation of the rhodopsin–mini-G_o_ complex was optimized to deliver the crystal structure. This was only the third crystal structure of a GPCR–G protein signaling complex. This approach can also be generalized for other membrane proteins and their complexes to facilitate sample preparation and structure determination.

## Introduction

Determining crystal structures of membrane proteins and their complexes has always been challenging due to difficulties in obtaining well-diffracting crystals. In contrast to soluble proteins, integral membrane proteins comprise a hydrophobic core that spans the cell membrane. To remove membrane proteins from the cell membrane into aqueous buffer, detergents have to be used to form a detergent-protein micelle, thus replacing the lipids around the hydrophobic core of membrane proteins. Stability, activity and integrity of membrane proteins are directly dependent on the chemical and structural properties of the detergent^1^, and the detergent's properties also determine the size of the micelle. A large detergent micelle may occlude the hydrophilic surfaces of a small membrane protein, thus preventing crystallization due to the lack of crystal contacts when using vapor diffusion method. A small detergent micelle is advantageous for crystallography, but short chain detergents are usually harsher and therefore lead to destabilization and aggregation of the membrane protein. Therefore, before crystallization, an additional detergent screening procedure is indispensable, typically targeting shorter detergents that still maintain protein stability.

G protein-coupled receptors (GPCRs) are integral membrane proteins containing seven transmembrane a-helices. GPCRs exist in two main states, either an inactive state stabilized by inverse agonists or antagonists, or an active state bound to an agonist and stabilized by a G protein, although it is likely that a multitude of sub-states exist between these two extremes. Structure determination of GPCRs initially focused on inactive states bound to inverse agonists and antagonists due to their higher stability than active states^2^. When GPCRs are activated upon agonist binding, the receptors are highly dynamic, and a cleft forms transiently on the cytoplasmic face of the receptor for G protein coupling. It is thought that this dynamism is why agonist-bound GPCRs are often more unstable than the inactive state. Therefore, it becomes essential to screen for detergents appropriate for the conformational state of the receptor under study, because it is likely that milder detergents will be required for studying an active state compared to an inactive state.

In this report, we use the visual GPCR, bovine rhodopsin^3^, and its complex with mini-G_o_ protein^4,5^ for the detergent screening experiments, representing the inactive state and active state, respectively. The detergent screening focused on the classical alkyl maltoside and glucoside detergents and the neopentyl glycol (NPG) detergents. In this context, a classical detergent is built from a sugar head group and an alkyl chain, while the NPG type detergents contains two identical classical detergents that are fused by a quaternary carbon at the interface between the sugars and the alkyl chains^6,7,8^.

**Figure 1:**
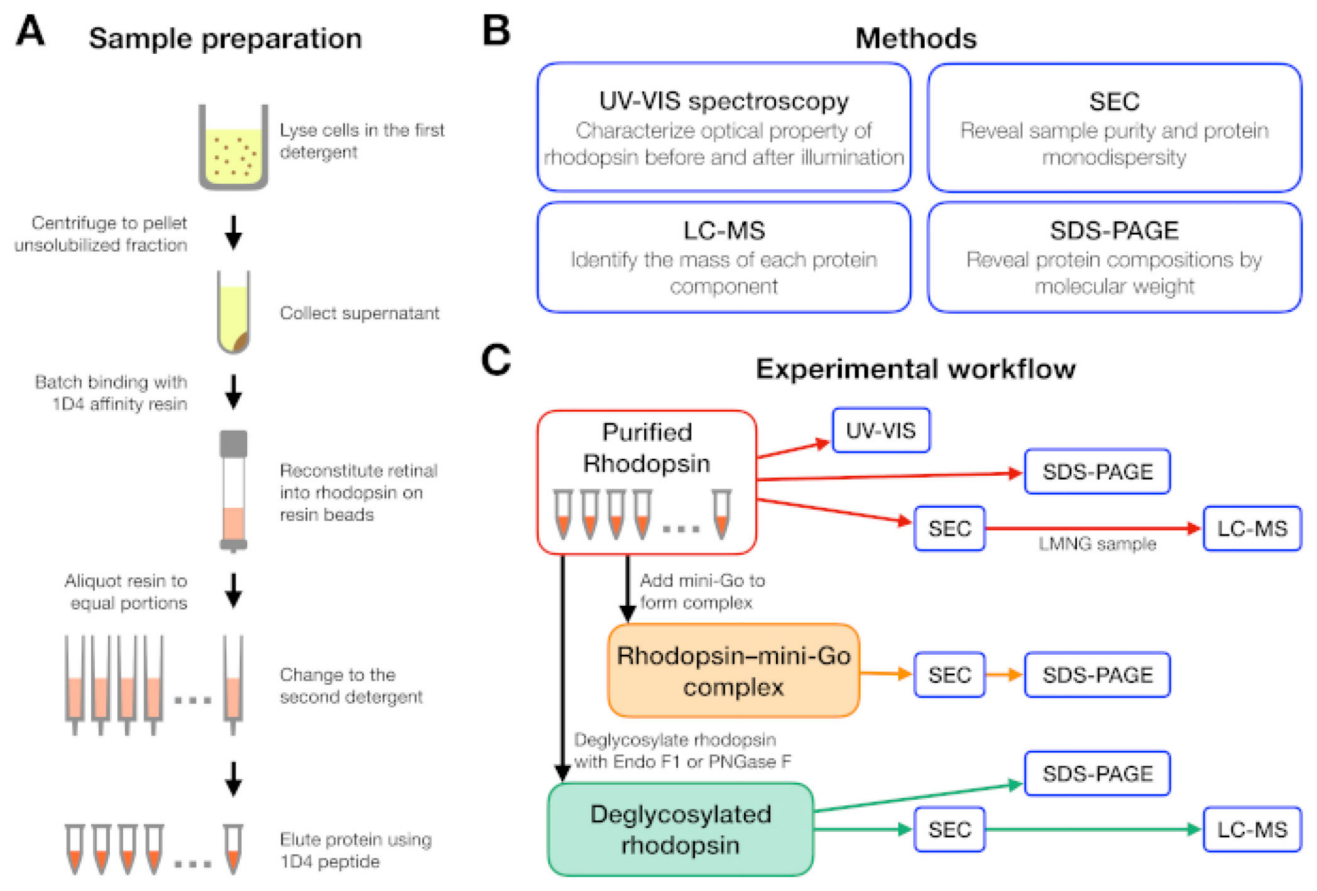
Sample preparation and characterization for detergent screening experiment. (**A**) Preparation of rhodopsin samples in different detergents during purification. (**B**) Methods used in the protocol: UV-VIS spectroscopy, size-exclusion chromatography (SEC), SDS-PAGE and liquid chromatography-mass spectrometry (LC-MS). (**C**) Experimental workflow for characterization of rhodopsin, rhodopsin–mini-G_o_, and deglycosylation product of rhodopsin. Please click here to view a larger version of this figure.

An experimental workflow was designed starting from the purification of rhodopsin in different detergents, followed by formation of the rhodopsin–mini-G_o_ complex and ending with the characterization of the complex using several methods (Figure 1). For the inactive state of rhodopsin, reconstitution of the light sensitive ligand 9-cis retinal was monitored by ultraviolet-visible (UV-VIS) spectroscopy. The spectrum reveals the physicochemical state of the retinal and is indicative of its environment in the retinal binding pocket of rhodopsin. Size exclusion chromatography (SEC) was employed to assess monodispersity of purified rhodopsin as well as formation of the rhodopsin–mini-G_o_ complex. As SEC differentiates protein molecules by their size and shape, aggregated protein population can be identified as they elute in the void volume. To confirm complex formation, fractions from SEC were assessed by sodium dodecyl sulfate-polyacrylamide gel electrophoresis (SDS-PAGE) to confirm the presence of both rhodopsin and mini-G_o_.

Another factor that needs to be considered is post-translational modifications (PTM) on the membrane proteins. PTM such as N-glycosylation are often observed on eukaryotic membrane proteins produced in mammalian and insect cell expression systems. A limited N-glycosylation strain of the human embryonic kidney 293 (HEK293) cells was developed by deletion of the gene encoding N-acetylglucosaminyltransferase I (GnTI), resulting in homogenous N-glycosylation by GlcNAc_2_Man_5_ at the consensus site Asn-X-Ser/Thr. Although N-glycosylation can be prevented by mutating an amino acid residue in the consensus site, this may also alter the function of the protein or the efficiency of folding. In bovine rhodopsin, mutation of the N-glycosylated residue Asn15 leads to incorrect folding and reduced G protein activation^9,10^. The rhodopsin used in this report was expressed in HEK 293 GnTI-deficient cell line. However, SDS-PAGE showed the presence of two species of rhodopsin. This heterogeneity could prevent crystal formation and therefore deglycosylation using peptide-N-glycosidase F (PNGase F) and endoglycosidase F1 (Endo F1) was tested. The deglycosylated product was characterized by SDS-PAGE and liquid chromatography-mass spectrometry (LC-MS) to identify the level of glycosylation and its homogeneity.

## Protocol

NOTE: This protocol for detergent screening is detailed for 30 g of HEK293 cell pellet as starting material.

### 1 Materials, chemicals and reagents

NOTE: All solutions are prepared using analytical grade reagents and ultrapure water, which is purified from deionized water to reach a resistivity of 18.2 MΩ∙cm at 25 °C.

1. Buffer stock solutions
  1. Prepare 10x phosphate buffered saline (10x PBS).
  2. Prepare HEPES buffer: 1 M, titrated to pH 7.5 with NaOH.
  3. Prepare 5 M NaCl.
  4. Prepare 2 M MgCl_2_.
    NOTE: All stock solutions are passed through a 0.22 μm filter to maintain their sterility.
2. Detergent stock solutions
  1. Prepare dodecyl maltoside (DDM), 10% (w/v).
  2. Prepare decyl maltoside (DM), 10% (w/v).
  3. Prepare 6-cyclohexyl-hexyl maltoside (Cymal-6), 10% (w/v).
  4. Prepare 5-cyclohexyl-pentyl maltoside (Cymal-5), 10% (w/v).
  5. Prepare nonyl glucoside (C9G), 10% (w/v).
  6. Prepare lauryl maltose neopentyl glycol (LMNG), 5% (w/v).
  7. Prepare decyl maltose neopentyl glycol (DMNG), 10% (w/v).
  8. Prepare cymal-6 neopentyl glycol (C6NG), 10% (w/v).
  9. Prepare cymal-5 neopentyl glycol (C5NG), 10% (w/v).
  10. Prepare octyl glucose neopentyl glycol (OGNG), 10% (w/v).
    NOTE: For 10% detergent stock solution, dissolve 1 g of detergent powder in ultrapure water with gentle rocking, and then adjust the final volume to 10 mL. Detergent stock solution should be kept at -20 °C for long-term storage and on ice whilst working.
    CAUTION: Bottled detergents are usually recommended to store at -20 °C freezer. The bottles containing detergent powder should be warmed to room temperature before opening. Detergent powder is hygroscopic, so temperature equilibration will prevent the formation of condensation that will wet the detergent.
3. Other chemicals and reagents
  1. Prepare 1D4 immunoaffinity agarose resin: 10 mL of the 50% slurry.
    NOTE: The 1D4 immunoaffinity agarose are the agarose beads linked with the monoclonal Rho1D4 antibody, which binds the last 9 amino acids of bovine rhodopsin TETSQVAPA as epitope. The 1D4 immunoaffinity agarose works as affinity purification material to capture proteins that contain a C-terminal 1D4 sequence. This purification material can be prepared^9,11^ or purchased.
  2. Prepare 9-cis retinal solution: 1 mM, dissolved in 100% ethanol.
    NOTE: Prevent light exposure to retinal during preparation and storage.
  3. Prepare 1D4 peptide (sequence TETSQVAPA): 800 μM, dissolved in water.
4. Buffers
  NOTE: All buffers are mixed from the stock solutions to the desired concentration. All buffers are chilled to 4 °C before use.
  1. Prepare Buffer A: PBS, 0.04% DDM.
  2. Prepare Buffer B: 20 mM HEPES pH 7.5, 150 mM NaCl, 0.04% DDM.
  3. Prepare Buffer C: 20 mM HEPES pH 7.5, 150 mM NaCl, and detergent at their working concentration listed in Table 1.
  4. Prepare Buffer D: 20 mM HEPES pH 7.5, 150 mM NaCl.
  5. Prepare Elution buffer: 20 mM HEPES pH 7.5, 150 mM NaCl, 80 μM 1D4 peptide, and detergent at their working concentration.
  6. Prepare SEC buffer: 20 mM HEPES pH 7.5, 150 mM NaCl, 0.025% DDM; filtrated through a 0.22 μm filter.
5. Solvent for LC-MS
  1. Prepare solvent A: acetonitrile containing 0.1% formic acid.
  2. Prepare solvent B: ultrapure water containing 0.1% formic acid.
  3. Prepare solvent C: iso-propanol.

### 2 Cell membrane solubilization and protein extraction

1. Thaw 30 g of HEK293 GnTI-cell pellet expressing the bovine rhodopsin mutant N2C/M257Y/D282C^3,9^ to room temperature, add 120 mL of 1x PBS buffer containing protease inhibitor cocktail and homogenize using a Dounce homogenizer or an electric homogenizer (13,000 rpm for 30 s). Collect the homogenized cell suspension in a beaker and adjust the volume to 150 mL.
  NOTE: 30 g of cell pellet is equivalent to 3 L of cell culture at 2 x 10^6^ cell/mL density.
2. Gently add 10% DDM to the homogenized cells to give a final concentration of 1.25%. Stir on ice for 1 h.
3. Centrifuge the cell lysate at 4 °C and 150,000 x *g* for 45 min to remove the unsolubilized debris.
4. Transfer the supernatant to a 500 mL bottle and add 10 mL of the 1D4 immunoaffinity agarose resin (50% slurry). Gently mix the solubilized cell lysate and resin for 4 h or overnight at 4 °C.
5. Load the lysate/resin mixture to an open column to collect the resin.
6. Wash the resin with 10 column volumes (CV) of the wash Buffer A.
  NOTE: The column volume is the volume of the packed (100%) agarose resin used. In this case, 1 CV is 5 mL.
7. Resuspend the resin with 2 CV of Buffer A.
  CAUTION: From step 2.8 onwards, steps that need to be carried out under dim red-light condition are labelled with "[Dark]" at the beginning of the description.
8. [Dark] Add 9-cis retinal to the resuspended resin to the final concentration of 50 μM. Gently mix at 4 °C for 4-16 h in the dark.
  NOTE: A shorter incubation time may lead to incomplete reconstitution of retinal.
9. [Dark] Remove flow through from the column. Wash resin with 20 CV Buffer A, followed by 15 CV Buffer B.
10. [Dark] Resuspend the resin in 2 CV Buffer B, and then divide the resin suspension equally to 10 10-mL disposal columns.
11. [Dark] Remove flow through from the column, and then resuspend the resin in 1 mL Buffer C. Incubate for 1 h at 4 °C.
12. [Dark] Repeat step 2.11.
13. [Dark] Remove flow through from the column, and then resuspend the resin in 0.8 mL Elution Buffer for each column. Gently mix for 2 h.
14. [Dark] Collect elution from the column into a 2 mL tube.
15. [Dark] Resuspend the resin in 0.7 mL of Elution Buffer for each column. Gently mix for 1 h.
16. [Dark] Collect elution from the column into the same tube.

### 3 UV-VIS spectroscopy

1. Prepare the spectrophotometer to cover the measurement range of 250-650 nm. Record the baseline using water or Elution Buffer.
2. [Dark] Load the eluted protein to the quartz cuvette. Measure the spectrum of the protein sample.
3. [Dark] Illuminate the protein directly in the cuvette for 2 min with light passed through a 495 nm long-pass filter.
4. Measure the spectrum of the illuminated sample.
5. Perform the same measurement for all the protein samples purified in the other 9 detergents, both dark and illuminated states.
6. Plot the curves (absorbance versus wavelength) in X-Y scatter chart.

### 4 Automated size-exclusion chromatography of rhodopsin and rhodopsin–mini-G_o_ complex

1. [Dark] Concentrate protein to 100 μL by centrifugation using a spin concentrator with a molecular weight cut-off (MWCO) of 30 kDa at 4 °C. Overconcentrated samples can be diluted using the flow through from the concentrator or Buffer C. To determine protein sample concentration, measure absorbance at 280 nm using a spectrophotometer.
  NOTE: From step 4.2 onwards, the experiment does not require a dark environment, and therefore samples can be prepared under normal light.
2. Prepare 100 μL rhodopsin at 0.7 mg/mL for each detergent condition.
3. Prepare 100 μL of rhodopsin (0.7 mg/mL) and mini-G_o_^4,12^ (0.2 mg/mL) mixture for each detergent condition. Supplement the mixture with 1 mM MgCl_2_. Illuminate the mixture with light from a 495 nm long-pass filter and incubate for 30 min.
4. Mount a 24 mL gel filtration column with a fractionation range of 10-600 kDa of a globular protein on a liquid chromatography purifier. Equilibrated the column with SEC buffer.
  NOTE: The liquid chromatography purifier is equipped with an autosampler, a multiple wavelength detector and a fraction collector.
5. Transfer the samples to the autosampler vials and place them in the sample tray. Program a method file to automate sequential SEC runs for each sample, with the autosampler loading 77 μL of the sample to the column, and the purifier eluting 24 mL of SEC buffer at a flow rate of 0.5 mL/min per run. Record the absorbance at 280 nm and 380 nm.
6. Collect the peak fractions of rhodopsin and rhodopsin–mini-G_o_ complex at the retention volume around 12.9 mL.
7. Analyze the left rhodopsin samples from step 4.2 and the peak fractions of rhodopsin–mini-G_o_ complex on 4-12% SDS-denaturing gradient gels with Coomassie blue staining.
8. Plot the elution chromatogram (A_280_ or A_380_ versus retention volume).

### 5 Deglycosylation and LC-MS study

1. For LC-MS study, only use the rhodopsin sample purified in LMNG detergent.
2. Prepare a 200 μL mixture of rhodopsin at 1 mg/mL and PNGase F^13^ at 0.01 mg/mL. Mix well and incubate at 4 °C overnight.
3. Prepare a 200 μL mixture of rhodopsin at 1 mg/mL and Endo F1^13^ at 0.01 mg/mL. Mix well and incubate at 4 °C overnight.
4. Analyze the digestion result by SDS-PAGE and Coomassie blue staining.
5. Concentrate untreated and Endo F1-treated rhodopsin samples and subject to SEC purification in Buffer D.
  NOTE: This is to prepare the sample with minimal quantity of detergent for LC-MS study. Buffer D does not contain any detergent, but due to the slow off-rate of LMNG from a membrane protein^14^, rhodopsin will not aggregate.
6. Collect the peak fraction at retention volume around 12.9 mL. Concentrate to 1 mg/mL using a spin concentrator (MWCO 30 kDa).
7. Inject 10 μg of the protein into a Reprosil 200 C18-AQ column and elute the column using the linear gradient method with the solvent composition and settings listed in Table 2. The flow is split to 25% for mass spectrometer and 75% for UV detection.

## Representative Results

The experimental workflow for sample preparation and analysis is summarized in Figure 1. Using open columns for small-scale affinity purification allowed us to prepare samples in many different detergent conditions in parallel (Figure 1A). Such a small-scale purification set-up yielded sufficient protein for further analyses using UV-VIS spectroscopy, SEC and SDS-PAGE (Figure 1B-C).

### UV-VIS spectroscopy revealed rhodopsin stability

**Figure 2:**
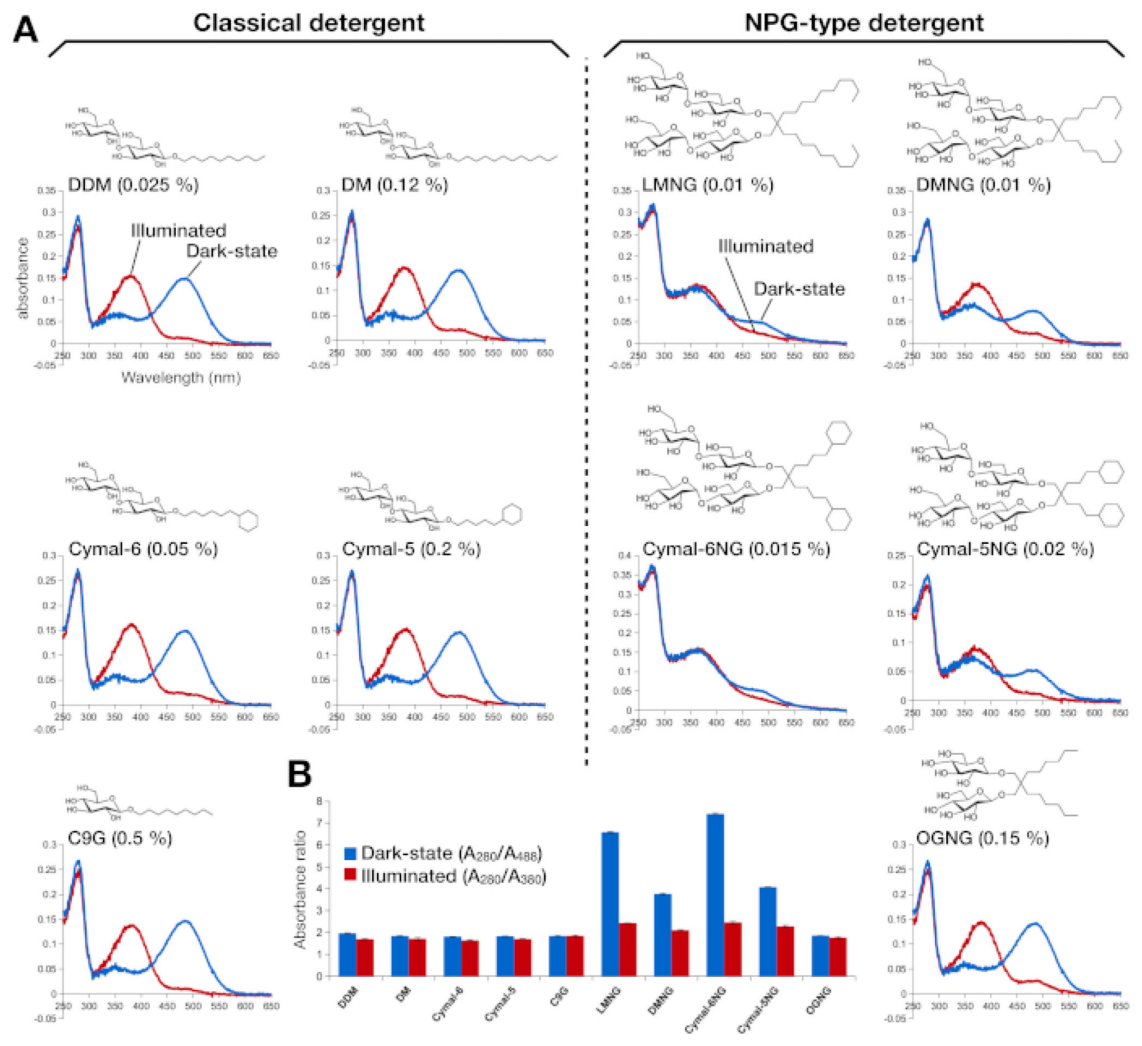
UV-VIS spectroscopy of rhodopsin. (**A**) UV-VIS spectra of rhodopsin. The spectra of the dark-state, 9-cis retinal-bound rhodopsin are shown in blue curves. After illumination, 9-cis retinal is deprotonated and isomerizes into all-trans retinal, and the spectra of illuminated rhodopsin are shown as red curves. The chemical structure of each detergent is shown as an inset. (**B**) The ratios of A_280_/A_488_ (blue bar) and A_280_/A_380_ (red bar) depict the stability of rhodopsin in the dark state and light state, respectively. Please click here to view a larger version of this figure

Stability of the retinal-reconstituted rhodopsin was assessed by its optical absorbance (Figure 2). In the dark state, 9-cis retinal is covalently linked to Lys296 as a protonated Schiff base. After illumination, the 9-cis retinal is isomerized to the all-trans isoform and the Schiff base link is deprotonated. The protonated 9-cis retinal gives an absorption peak at 488 nm, while the deprotonated all-trans retinal has a peak at 380 nm. The UV-VIS spectra of rhodopsin in DDM showed the typical absorption of 9-cis retinal-bound and light-activated rhodopsin, where a blue shift of 108 nm with roughly the same optical density was clearly observed (Figure 2A, **upper left panel**). When rhodopsin is destabilized, and then the binding pocket for retinal changes, which results in retinal deprotonation and possibly dissociation. If this happens, and then the spectrum shows the contribution from deprotonation as well as the free form of retinal^15^. Therefore, we determined the efficiency of retinal reconstitution into rhodopsin by the absorbance ratio between the protein (280 nm) and the retinal (488 nm for protonated 9-cis retinal, 380 nm for deprotonated all-trans retinal) (Figure 2B). Rhodopsin samples purified in the classical detergents (DDM, DM, Cymal-6, Cymal-5, C9G) show the same optical profile. However, the samples purified in the NPG detergents (LMNG, DMNG, Cymal-6NG, Cymal-5NG) show optical profiles suggesting a sub-optimal binding environment for retinal except for the OGNG sample, which gave the same optical profile as the DDM sample.

### Size-exclusion chromatography showed sample purity and protein monodispersity

**Figure 3:**
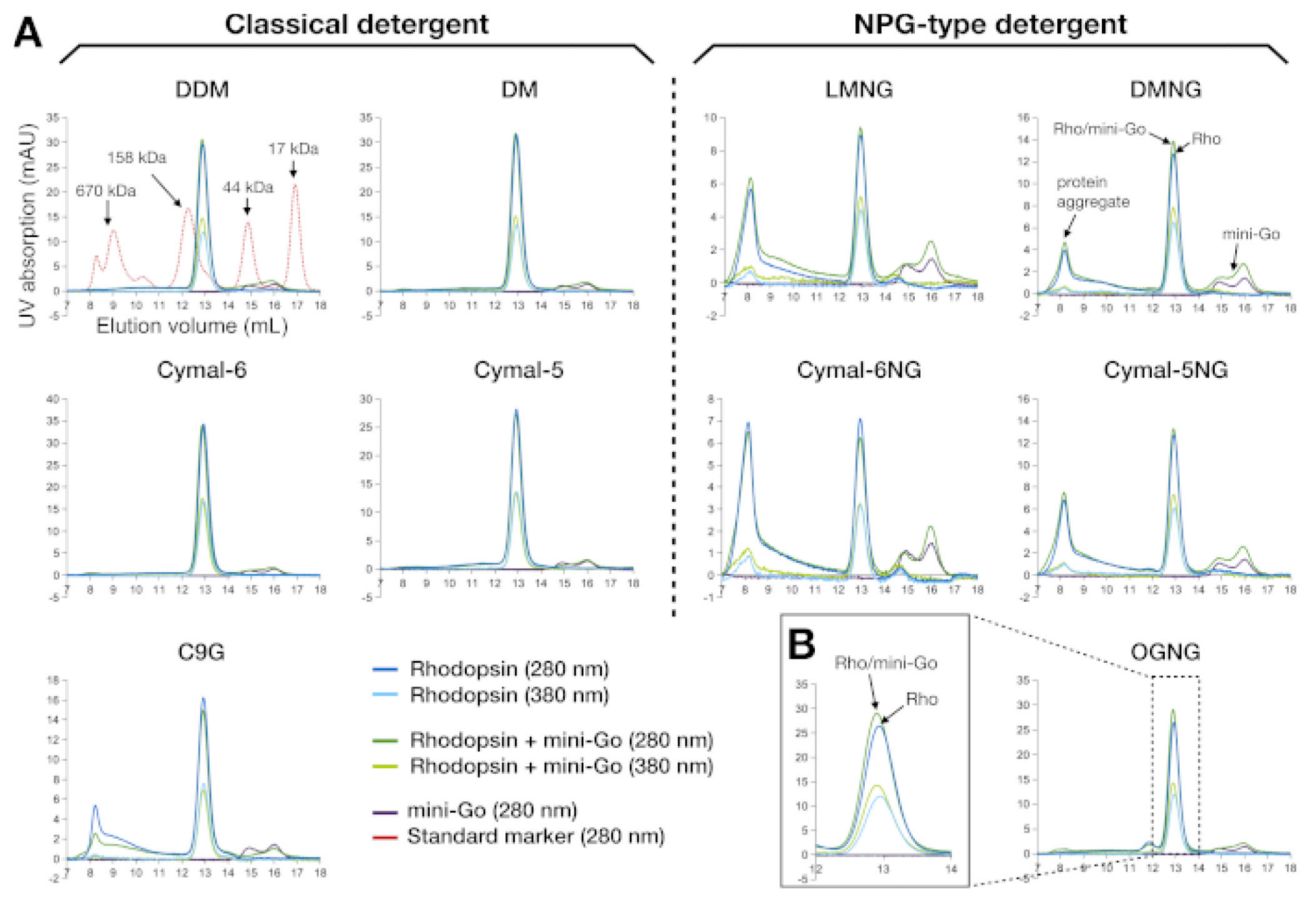
Size-exclusion chromatography profiles of rhodopsin and rhodopsin–mini-G_o_ complex purified in 10 different detergents. (**A**) The left panel shows the SEC profiles of samples purified in the classical detergents. The right panel represents the SEC profiles of samples purified in the NPG type detergents. The profile of the standard marker proteins is shown as overlay together with the DDM sample. The interpretation of the peak profiles is shown for DMNG, with the ideal scenario (no aggregates) seen for DDM, DM, Cymal-6, Cymal-5 and OGNG. (**B**) The magnified profile of the OGNG sample in the retention volume of 12-14 mL. All samples were analyzed using a Superdex200 Increase 10/300 GL column. Please click here to view a larger version of this figure.

SEC is an efficient and robust analytical tool for evaluating protein samples during preparation and screening. It validates sample purity from the previous purification step as well as the monodispersity of the protein molecules. For rhodopsin and its mini-G_o_ complex, the sample quality was interpreted from the absorption curves at 280 nm and 380 nm (Figure 3A). The 280 nm traces showed the presence of protein, and the 380 nm trace showed the presence of retinal. Any signals appearing in the void volume (around 8 mL when using this column) were attributed to protein aggregates. Therefore, the results showed that samples prepared in the classical detergents were in a monodisperse state except for C9G, where some portion of aggregate appeared. In contrast, samples prepared using the NPG-type detergents contained much more aggregates than the C9G sample; LMNG and Cymal-6NG led to the most aggregate formation, but less aggregates were observed in DMNG and Cymal-5NG. The exception was OGNG, which showed a similar profile to DDM. Protein aggregates eluting at the void volume also had poorer retinal occupancy, as shown by the A_280_/A_380_ ratio that had increased in comparison to the peak at the retention volume of ~12.9 mL corresponding to 135 kDa. Another feature we observed was that both rhodopsin and rhodopsin–mini-G_o_ eluted around the same retention volume (Figure 3B). This is unsurprising, because the apparent molecular weight of detergent-bound rhodopsin was 120 kDa and that of rhodopsin–mini-G_o_ 144 kDa. We therefore could not ascertain complex formation merely from the SEC data, so SDS-PAGE was used to further analyze the SEC-purified sample.

### SDS-PAGE confirmed complex formation

**Figure 4:**
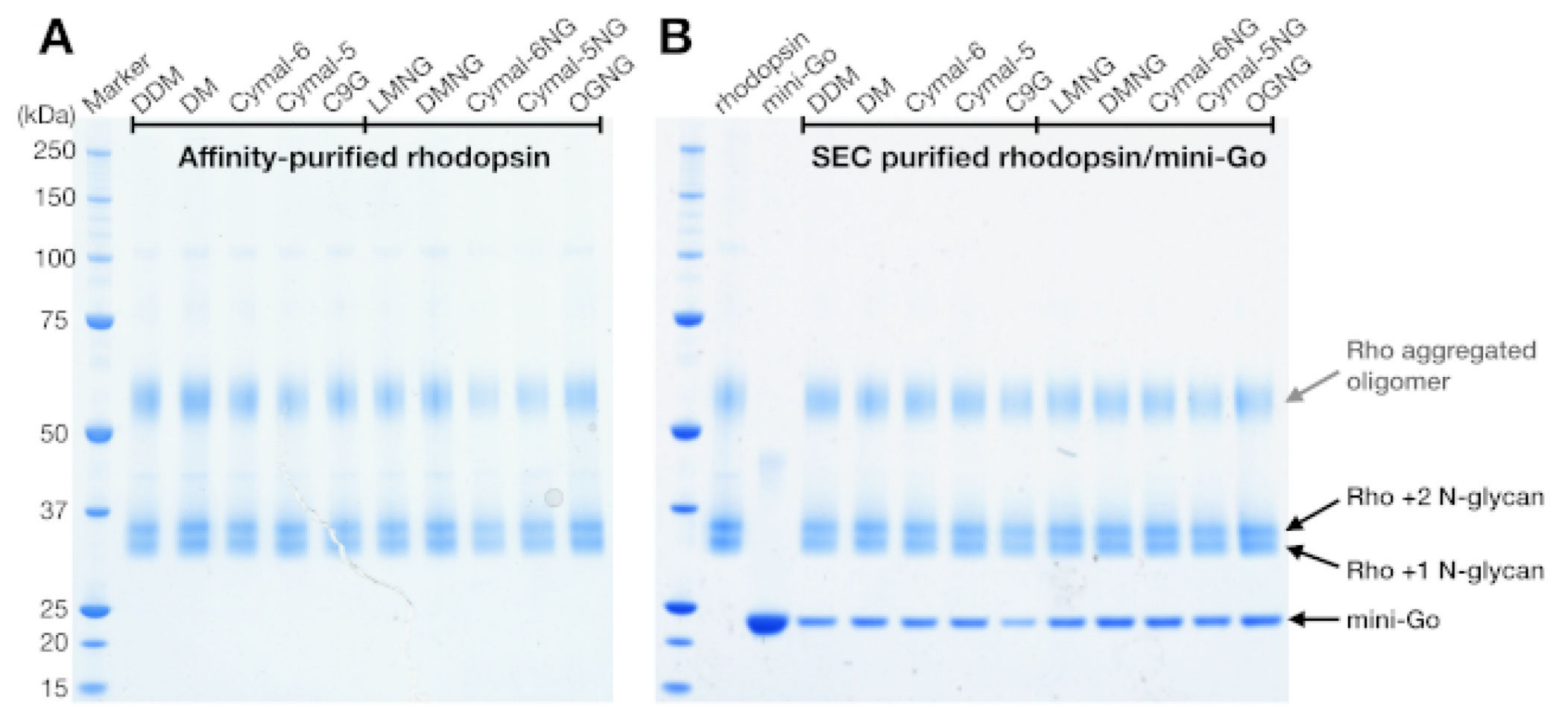
SDS-PAGE analysis of rhodopsin and rhodopsin/mini-G_o_ complex. (**A**) Rhodopsin samples purified in detergents. The smeared band above 50 kDa is attributed to the aggregated rhodopsin oligomers induced by the SDS-PAGE sample buffer. (**B**) SEC-purified samples of rhodopsin/mini-G_o_ complex. Rhodopsin with 1 and 2 N-glycan and mini-G_o_ are depicted. Please click here to view a larger version of this figure.

SDS-PAGE is a standard method to identify the protein components in a sample. Concentrated rhodopsin (prior to SEC purification) were analyzed by SDS-PAGE to confirm its purity, and showed two bands near 37 kDa and a smeared band above 50 kDa (Figure 4A). The lower two bands were later confirmed to have different N-glycosylation states. The band above 50 kDa was interpreted as aggregated rhodopsin oligomers induced by the SDS-PAGE sample buffer because these aggregates were not observed in SEC or any other detection methods. As SEC data could not confirm complex formation, the SEC eluted fractions from rhodopsin–mini-G_o_ samples were analyzed using SDS-PAGE. The SDS-PAGE showed protein bands of both rhodopsin and mini-G_o_ in all the detergent conditions, suggesting the complex was formed regardless of the choice of detergent (Figure 4B).

### LC-MS spectrometry identified the N-glycosylation pattern in rhodopsin

**Figure 5:**
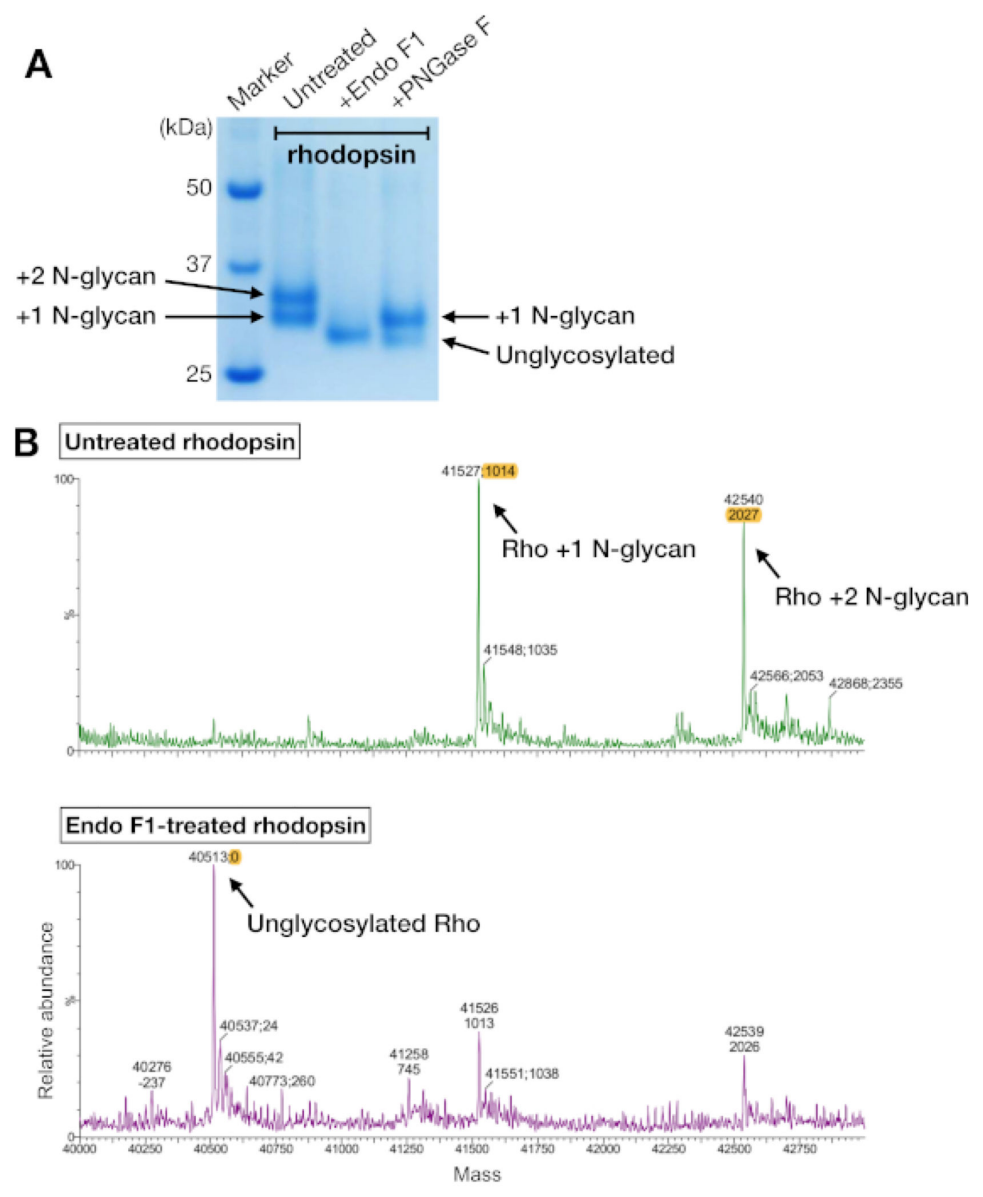
Identification of glycosylation in rhodopsin. (**A**) SDS-PAGE analysis of deglycosylated rhodopsin using PNGase F and Endo F1. (**B**) LC-MS spectra of rhodopsin without (upper panel) and with deglycosylation by Endo F1 (lower panel). For preparing the rhodopsin–mini-G_o_ complex for crystallization, we chose Endo F1 over PNGase F because Endo F1 delivered one single homogeneous species of rhodopsin. Please click here to view a larger version of this figure.

Rhodopsin samples from both affinity purification and SEC showed two protein bands that migrated with an apparent molecular weight of about 37 kDa on an SDS-PAGE gel, which could not be separated by SEC when using a 24-mL column. Different patterns of N-glycosylation on the heterologously-expressed rhodopsin from HEK 293 GnTI^-^cells was the most likely explanation. Therefore, two enzymes, PNGase F and Endo F1, were tested for their ability to deglycosylate rhodopsin. From the SDS-PAGE data, Endo F1 reduced the molecular weight of both protein bands into a single product, while PNGase F digestion still gave two populations (Figure 5A). The undigested and Endo F1-treated samples were analyzed using LC-MS spectrometry to identify the masses of different species. The data showed that rhodopsin produced in HEK 293 GnTI-cells contained either one or two N-glycans, with a difference in mass of 1014±1 Da. Endo F1-treated rhodopsin did not contain any N-glycans and had a mass difference of 2027±1 Da compared to rhodopsin containing two N-glycans. These results are consistent with the absence of the enzyme N-acetylglucosaminyltransferase I in the cell line used to express rhodopsin, which results in all N-glycans having the structure GlcNAc_2_Man_5_, (mass 1014 Da).

## Discussion

The success in protein crystallization strongly relies on the protein sample, especially membrane proteins and their complexes due to the complication caused by detergents. This report demonstrates detergent screening and evaluation of sample quality for GPCR–mini-G protein signaling complexes. A variety of methods have been widely used to study the biochemical property of membrane proteins, for example, thermostability assay using fluorescent dyes^16,17^, binding assay to detect complex formation by measuring the change in tryptophan fluorescence signal^18^ or the resonance energy transfer with biosensors^19^. However, the chemical environments used in those methods are quite different from those for preparing a crystallization sample, either proteins are at a thousand-fold lower concentration for fluorescence-based measurement, or proteins are embedded in lipid bilayers or in one fixed detergent condition. In this protocol, the used methods are also standardized in large-scale sample preparation before crystallization. Therefore, the optimized parameters can be easily transferred for crystallization-scale preparation without further major screening and optimization.

The aim of this protocol is to optimize the preparation of a stable and homogeneous GPCR–mini-G protein complex for vapor diffusion crystallization and structure determination by X-ray crystallography. The protocol integrates a set of methods to qualitatively evaluate the impact of detergent and deglycosylation during preparation of the rhodopsin–mini-G_o_ complex. Rhodopsin at inactive state and light-activated state bound with and without the transducin peptide has been crystallized when purified in the detergents octyl glucoside (C8G)^20,21,22^ and C9G^23,24^. As the rhodopsin–mini-G_o_ complex purified in C8G and C9G did not yield crystals (data not shown), we then explored a wider range of other detergents using the strategy described (Figure 1). By taking the advantage of the light sensitivity of rhodopsin, we could very well follow the reconstitution of retinal at wavelengths other than 280 nm. In both UV-VIS spectroscopy and SEC, we detected retinal at either 380 nm or 488 nm. However, most membrane proteins do not have such a convenient chromophore to follow functionality during purification. Other options would be to make a ligand detectable by adding a light-detectable chromophore or by using radioligand-binding and thermal shift assays^25^.

Rhodopsin has a molecular weight of 40 kDa. Due to the mass of detergent it binds, its apparent molecular weight on SEC is about 120 kDa. It is thus no surprise that the binding of mini-G_o_ (24 kDa) was not easily detected on SEC, as this would necessitate differentiation of proteins with apparent masses of 120 kDa and 144 kDa. Analysis of SEC fractions by SDS-PAGE was therefore used to confirm sample purity and complex formation. Even if SEC profiles show a clear shift upon complex formation, it is still recommended to perform SDS-PAGE analysis to confirm the complex formation with correct binding partners rather than other co-purified protein contaminants.

Both rhodopsin and mini-G_o_ were purified in milligram quantities, which allowed the use of low sensitivity detection of the complexes, such as UV-VIS absorption during SEC and Commassie Blue staining of SDS-PAGE gels. Where samples are limited, more sensitive detection should be used, such as an LC purifier equipped with a fluorescence detector to trace tryptophan signals from the protein (280 nm excitation, 350 nm emission) and silver-staining for SDS-PAGE gels. Another approach would be to fuse a fluorescent protein, such as green fluorescence protein (GFP) to the protein of interest, which would also allow detection even during protein expression^26^ but it should be removed before crystallization.

It is essential to ensure that the purified protein is also free of heterogeneity arising from variable PTMs. In the case described here, the two populations of rhodopsin observed on SDS-PAGE gels were characterized as having either one or two N-glycans. Variable modification of a protein would potentially prevent the formation of well-diffracting crystals, so we therefore deglycosylated rhodopsin. The endoglycosidase Endo F1 was the most effect endoglycosidase tested and treatment led to a single species of unglycosylated receptor, while PNGase F only partially removed the glycans on rhodopsin and resulted in a mixture of rhodopsin fully unglycosylated or with one N-glycan remained. Rhodopsin without deglycosylase treatment has been successfully crystallized^3,27,28^, and the N-glycan on rhodopsin Asn15 is important to form crystal contact in those cases. In the case of rhodopsin–mini-G_o_, it is necessary to remove N-glycans by Endo F1 to obtain crystals. There is no standardized rule to deglycosylate proteins of interest before crystallization, but removal of heterogenous PTMs should be considered when proteins fail to crystallize after extensive crystallization trials.

The data and methodology described here guided us to choose OGNG as the most preferred detergent for crystallization of the rhodopsin–mini-G_o_ complex due to its small micelle size and its ability to stabilize the complex. We also used Endo F1 to ensure the purified rhodopsin was a homogeneous species. Crystals were subsequently obtained and we determined the crystal structure to ~3.1 Å^4^, which was only the third crystal structure of a GPCR–G protein signaling complex^14,29^.

For membrane proteins bound with and without a partner protein, they should be considered as two different proteins. A protein at different functional states has different conformations and is at different energy level. Therefore, it is recommended to optimize the preparation protocol for each functional state as the parameter for the inactive-state may not be fully transferrable to the activated state. Also, not to mention the change in the protein property complicated by binding a partner protein. The protocol uses methods that are standardized for preparing a crystallization sample to prepare inactive membrane protein in different detergents, followed by protein activation and complex formation, and to characterize protein quality. Thus, this protocol can easily be generalized to other membrane proteins and their complexes for structural studies with minor modification.

## Figures and Tables

**Table 1:** Buffer C detergent concentrations.

Detergent	Working concentration (%)	Critical micelle concentration (%)
DDM	0.025	0.0087
DM	0.12	0.087
Cymal-6	0.05	0.028
Cymal-5	0.2	0.12
C9G	0.5	0.2
LMNG	0.01	0.001
DMNG	0.01	0.0034
Cymal-6NG	0.015	Not available; should be lower than 0.056
Cymal-5NG	0.02	0.0056
OGNG	0.15	0.058

**Table 2:** Column elution parameters.

Time (min)	Solvent A (%)	Solvent B (%)	Solvent C (%)	Flow rate (ml/min)
0	0	95	5	0.5
1	0	95	5	0.5
5	20	75	5	0.6
25	85	10	5	0.6
26	90	5	5	0.6
30	90	5	5	0.6

## References

[1] Tate CG (2010). Practical considerations of membrane protein instability during purification and crystallisation. Methods in Molecular Biology (Clifton, N.J.).

[2] Lebon G, Bennett K, Jazayeri A, Tate CG (2011). Thermostabilisation of an agonist-bound conformation of the human adenosine A(2A) receptor. Journal of Molecular Biology.

[3] Deupi X (2012). Stabilized G protein binding site in the structure of constitutively active metarhodopsin-II. Proceedings of the National Academy of Sciences.

[4] Tsai C-J (2018). Crystal structure of rhodopsin in complex with a mini-G o sheds light on the principles of G protein selectivity. Science Advances.

[5] Carpenter B, Tate CG (2016). Engineering a minimal G protein to facilitate crystallisation of G protein-coupled receptors in their active conformation. Protein Engineering Design and Selection.

[6] Chae PS (2010). Maltose-neopentyl glycol (MNG) amphiphiles for solubilization, stabilization and crystallization of membrane proteins. Nature Methods.

[7] Loll PJ (2014). Membrane proteins, detergents and crystals: what is the state of the art?. Acta Crystallographica Section F Structural Biology Communications.

[8] Chae PS (2013). Glucose-neopentyl glycol (GNG) amphiphiles for membrane protein study. Chemical communications (Cambridge, England).

[9] Standfuss J, Xie G, Edwards PC, Burghammer M, Oprian DD, Schertler GFX (2007). Crystal structure of a thermally stable rhodopsin mutant. Journal of Molecular Biology.

[10] Kaushal S, Ridge KD, Khorana HG (1994). Structure and function in rhodopsin: the role of asparagine-linked glycosylation. Proceedings of the National Academy of Sciences of the United States of America.

[11] Molday LL, Molday RS (2014). 1D4: a versatile epitope tag for the purification and characterization of expressed membrane and soluble proteins. Methods in Molecular Biology (Clifton, N.J.).

[12] Carpenter B, Tate CG (2017). Expression and Purification of Mini G Proteins from Escherichia coli. Bio-Protocol.

[13] Grueninger-Leitch F, D'Arcy A, D'Arcy B, Chène C (1996). Deglycosylation of proteins for crystallization using recombinant fusion protein glycosidases. Protein Science.

[14] Rasmussen SGF (2011). Crystal structure of the β 2 adrenergic receptor-Gs protein complex. Nature.

[15] Loginova MY, Rostovtseva YV, Feldman TB, Ostrovsky MA (2008). Light damaging action of all-trans-retinal and its derivatives on rhodopsin molecules in the photoreceptor membrane. Biochemistry (Moscow).

[16] Alexandrov AI, Mileni M, Chien EYT, Hanson MA, Stevens RC (2008). Microscale Fluorescent Thermal Stability Assay for Membrane Proteins. Structure.

[17] Sonoda Y (2011). Benchmarking Membrane Protein Detergent Stability for Improving Throughput of High-Resolution X-ray Structures. Structure.

[18] Maeda S (2014). Crystallization scale preparation of a stable GPCR signaling complex between constitutively active rhodopsin and G-protein. PloS One.

[19] Boute N, Jockers R, Issad T (2002). The use of resonance energy transfer in high-throughput screening: BRET versus FRET. Trends in Pharmacological Sciences.

[20] Singhal A, Guo Y, Matkovic M, Schertler G, Deupi X, Yan ECY (2016). Structural role of the T 94 I rhodopsin mutation in congenital stationary night blindness. EMBO Report.

[21] Choe H-W (2011). Crystal structure of metarhodopsin II. Nature.

[22] Mattle D (2018). Ligand channel in pharmacologically stabilized rhodopsin. Proceedings of the National Academy of Sciences of the United States of America.

[23] Okada T, Fujiyoshi Y, Silow M, Navarro J, Landau EM, Shichida Y (2002). Functional role of internal water molecules in rhodopsin revealed by X-ray crystallography. Proceedings of the National Academy of Sciences of the United States of America.

[24] Blankenship E, Vahedi-Faridi A, Lodowski DT (2015). The High-Resolution Structure of Activated Opsin Reveals a Conserved Solvent Network in the Transmembrane Region Essential for Activation. Structure.

[25] Magnani F (2016). A mutagenesis and screening strategy to generate optimally thermostabilized membrane proteins for structural studies. Nature Protocols.

[26] Kawate T, Gouaux E (2006). Fluorescence-detection size-exclusion chromatography for precrystallization screening of integral membrane proteins. Structure (London, England: 1993).

[27] Standfuss J (2011). The structural basis of agonist-induced activation in constitutively active rhodopsin. Nature.

[28] Singhal A (2013). Insights into congenital stationary night blindness based on the structure of G90D rhodopsin. EMBO reports.

[29] Carpenter B, Nehmé R, Warne T, Leslie AGW, Tate CG (2016). Structure of the adenosine A(2A) receptor bound to an engineered G protein. Nature.

